# Amyand's hernia: a case report and literature review

**DOI:** 10.3389/fped.2025.1637375

**Published:** 2025-07-09

**Authors:** Li Zhang, Zihan Chen, Yanxiang Fu, Qi Zhang, Hailong Hu

**Affiliations:** Department of General Surgery, Yellow River Sanmenxia Hospital, Sanmenxia, China

**Keywords:** Amyand's hernia, case report, literature review, laparoscopy, hernia

## Abstract

Amyand's hernia (AH), an extremely rare form of inguinal hernia, particularly those cases where the hernial sac contains an inflamed, edematous, or perforated appendix, is infrequently encountered. This report details the case of a 6-year-old boy who presented with an irreducible, painful mass in the right inguinal region of unknown etiology. A CT scan indicated right inguinal hernia, and a blood routine test revealed elevated white blood cell counts. The patient was diagnosed with right inguinal hernia (AH) and acute appendicitis. Emergency laparoscopic high ligation of the inguinal hernia sac and appendectomy were performed. A postoperative pathological examination confirmed acute simple appendicitis and periappendicitis. The patient recovered uneventfully and was discharged 5 days after surgery. With a 6-month follow-up, no recurrence was observed, and the patient remains under follow-up. In conclusion, AH is a rare condition, and laparoscopic high ligation of the hernial sac is the primary treatment approach. During surgery, careful identification of the hernia contents is essential. Comprehensive preoperative assessment, precise surgical techniques, and standardized intraoperative and postoperative management play a vital role in minimizing complications and reducing the risk of postoperative recurrence.

## 1 Introduction

Inguinal indirect hernia in children is a common congenital malformation in pediatric surgery, with an incidence of 0.8%–5.0% in full-term infants and 30% in premature and low-birth-weight infants. The incidence of Amyand's hernia (AH) is approximately 1% in all children, and is more frequent in boys, which may be attributed to the anatomical predisposition of the appendix to herniate through the inguinal canal (1, 2). When the content of the inguinal indirect hernia in children is a normal or ischemic appendix, it is called AH, which was first reported by the British surgeon Claudius Amyand in 1735 (3). This type of hernia is not common clinically, especially cases where the hernial sac contains an inflamed and edematous or perforated appendix, with an incidence of 0.06%–0.07%. Also, due to the unclosed processus vaginalis in children, the incidence of AH is three times that in adults (3, 4). Here, we present a case of AH and review the relevant literature.

## 2 Case/case series presentation

**Figure 1:**
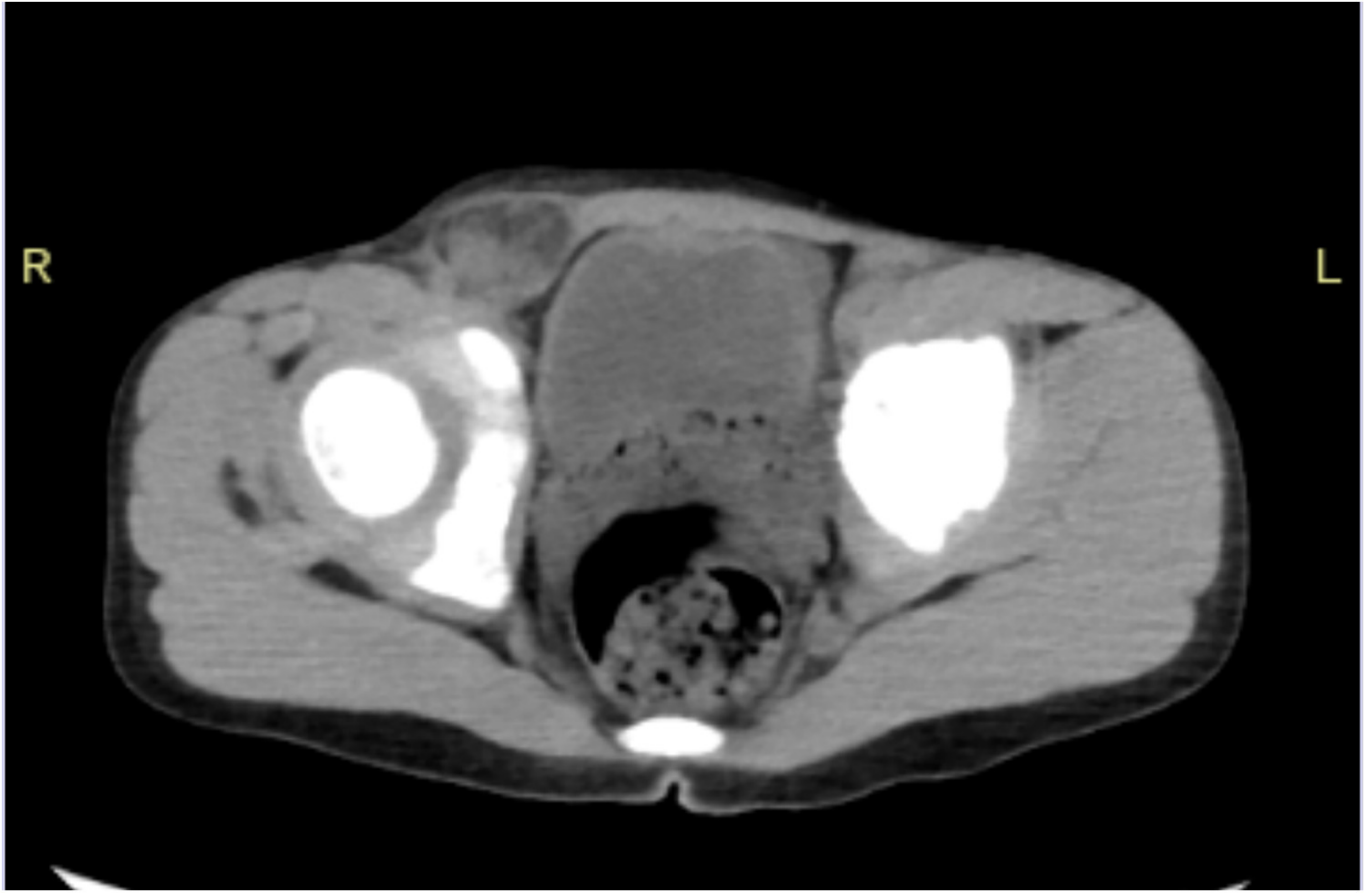
CT of the lower abdomen showed a part of the small intestine and fat in the right inguinal region herniated into the right hernial sac.

In December 2024, our hospital's emergency department admitted a 6-year-old boy. The patient presented with a painful mass in the right inguinal region that could not be reduced for 8 h. After admission, a physical examination showed that the patient's abdomen was flat and soft, without obvious tenderness, rebound tenderness, or muscle tension. There was deep tenderness in the right inguinal region near the pubic symphysis, without rebound tenderness. A mass of about 3 cm × 3 cm in size was visible in the right inguinal region, with no redness or swelling on the surface, positive for tenderness, and it could not be reduced. An auxiliary examination showed that the blood routine white blood cells were 10.34 × 10^9^/L (reference value: 3.5–9.5 × 10^9^/L) and neutrophils were 7.19 × 10^9^/L (reference value: 1.8–6.3 × 10^9^/L). A lower abdominal CT showed that a part of the small intestine and fat in the right inguinal region herniated into the right hernial sac, and no other abnormalities were found, suggesting right inguinal hernia (Figure 1). The child had a history of surgical treatment for “right inguinal hernia” in our hospital 4 years ago.

**Figure 2:**
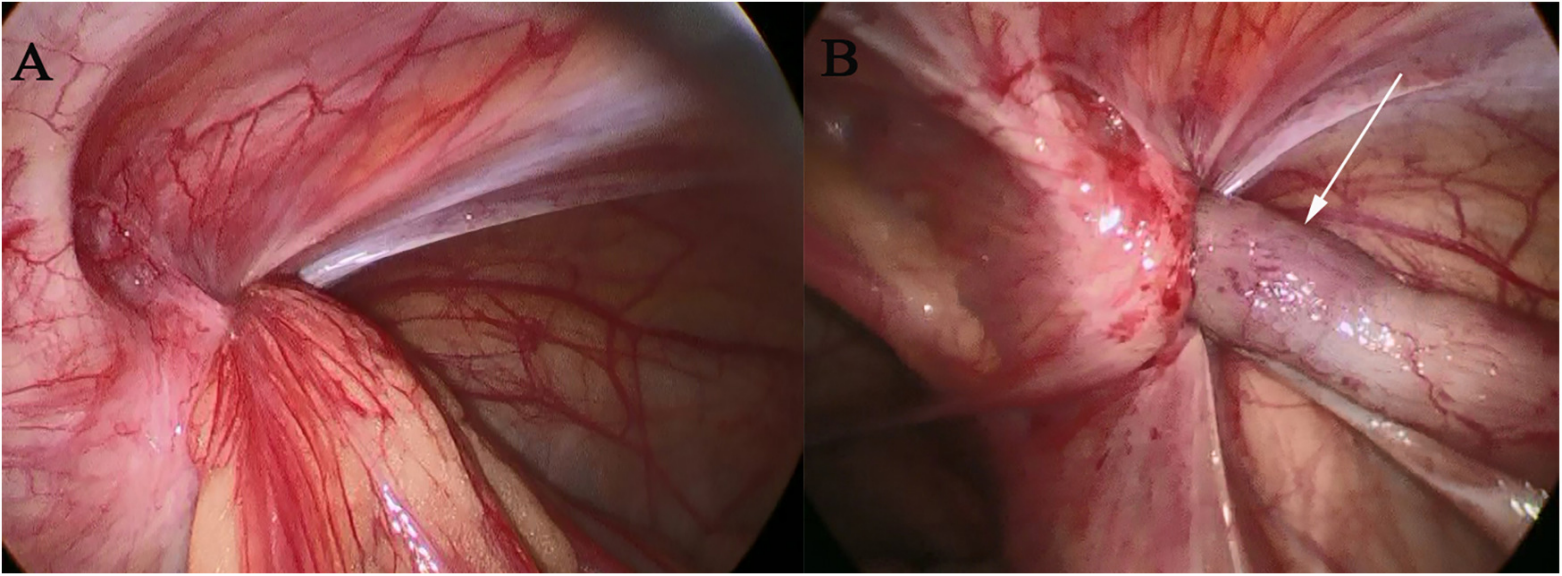
Intraoperative laparoscopic conditions: in (**A**), the hernia sac opening can be seen, and in (**B**), the hernia contents—the appendix, indicated by the white arrow.

After the anesthesiology department jointly evaluated the patient's condition, right laparoscopic high ligation of the hernial sac of the inguinal hernia and appendectomy were performed under general anesthesia. After the success of anesthesia, the patient was placed in the supine position. The skin was routinely disinfected and covered with a sterile drape. A small arc-shaped incision was made under the umbilicus. A pneumoperitoneum needle was inserted into the abdominal cavity, and carbon dioxide gas was insufflated to create a pneumoperitoneum of 10 mmHg. The pneumoperitoneum needle was removed, and a trocar was inserted into the abdominal cavity. The inner core was removed, and a laparoscope was inserted. Exploration of the abdominal cavity showed that the omentum and appendix herniated into the defect of the right abdominal wall (Figure 2A). A 5 mm trocar was placed beside the rectus abdominis muscle at the umbilical level on the right side. The grasping forceps pulled the herniated tissue into the abdominal cavity (Figure 2B). After manual reduction, the pulled-back tissue showed that the appendix was red and swollen, and there seemed to be a diverticulum at the front end. There was no obvious bleeding in the omentum, and the hernial ring was obviously red and swollen, without obvious bleeding. Since the appendix was in an acutely edematous state, after discussion with the family members, it was decided to perform appendectomy. A 1 mm incision was made on the surface of the right internal ring. A puncture needle with a thread was inserted through the incision until it reached the peritoneal layer. The puncture needle with the thread passed closely along the peritoneum and introduced the ligation thread into the medial half-ring of the defect. The spring clip thread needle entered along the lateral half-ring of the defect and lifted the thread outside the abdominal wall. The ligation thread surrounded the defect opening for one week. The spermatic cord, vas deferens, and inferior epigastric vessels were protected, and the suture was tightened. Under laparoscopic monitoring, a 10 mm trocar was placed at the outer one-third between the umbilicus and the left anterior superior iliac spine, and a 5 mm trocar was placed at the midpoint between the umbilicus and the pubic symphysis. The defect opening was sutured again with 3–0 absorbable sutures. A part of the right medial umbilical ligament covered the defect opening satisfactorily. The patient was changed to a head-down and foot-up left lateral position. The abdominal effusion was aspirated with a suction device. The appendix was lifted with a grasping forceps, and the mesoappendix was processed with a harmonic scalpel until the root of the appendix. The root of the appendix was ligated with a No. 4 silk thread. The appendix was transected 0.5 cm away from the ligation line with a harmonic scalpel. The stump of the appendix was buried with 3-0 absorbable sutures. A drainage tube was placed beside the appendiceal area and led out from the right lower abdomen. It was checked that there was no bleeding in the abdominal cavity. The incision was sutured, the drainage tube was fixed, and the operation was completed. The intraoperative blood loss was about 10 ml, and the specimen was sent for a pathological examination.

**Figure 3:**
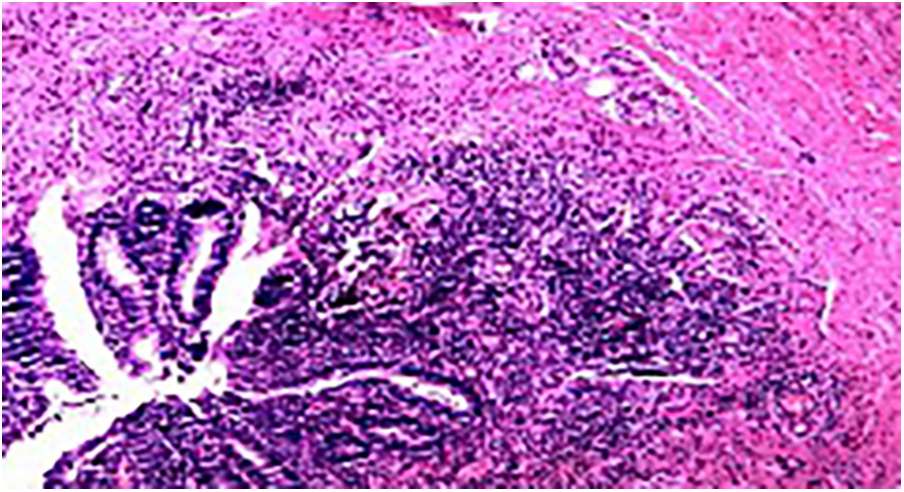
Pathological results of the appendix (H&E staining).

Postoperative pathology: Acute simple appendicitis, periappendicitis, and reactive hyperplasia of the surrounding lymph nodes (one piece) (Figure 3).

## 3 Discussion

AH is a special type of inguinal hernia, with the appendix as the content of the hernia; besides, cecum as a content of the inguinal hernia sac has also been documented; however, perforation of the cecum is a rare finding (5). Its incidence is low, and most of them are indirect hernias. According to the pathological condition of the appendix, it can be divided into four types: Type I, normal appendix; Type II, acute appendicitis with inflammation limited to the hernial sac; Type III, acute appendicitis complicated by peritonitis (intestinal necrosis, testicular necrosis, empyema of the hernial sac, etc.); Type IV, acute appendicitis complicated by other abdominal lesions outside the hernial sac, such as colon cancer, appendiceal neoplastic lesions, and so on. (6). Because of its low incidence, it is easy to be missed or misdiagnosed. An imaging examination is helpful for the diagnosis of AH. Color Doppler ultrasound can determine whether there is an appendix in the AH hernial sac through characteristic manifestations such as double-sided cord-like shadows of the appendix, communication with the cecum, thin wall, absence of mucosal folds, and immobility (7, 8). According to the literature report, the preoperative ultrasonic diagnostic coincidence rate of AH in children is 93.3%, but it is greatly influenced by the examiner's personal skills and experience. Sometimes, a further CT examination is required to confirm the diagnosis (9, 10). In recent years, more and more reports on the preoperative CT diagnosis of AH have been published, and it can also be used to distinguish inguinal indirect hernia, direct hernia, and femoral hernia before surgery. However, some scholars believe that when the clinical symptoms are relatively clear, imaging examination is unnecessary, and surgery should be performed immediately (11, 12).

At present, there is still some controversy about the treatment of AH, for example, whether a mesh can be placed. Some literature studies point out that placing a mesh may lead to postoperative infection (3). However, with the increase of research, according to the existing published literature, this does not seem to be the case. Placing a mesh does not increase the infection rate even in patients with acute appendicitis (13, 14). Another controversial issue focuses on whether prophylactic appendectomy should be performed. More and more scholars believe that the decision should be made according to the patient's age, the risk of recurrent acute appendicitis in a lifetime, and the size and anatomy of the appendix. Children have a high risk of recurrent appendicitis in their lifetime, and appendectomy is recommended, while the appendix in middle-aged or elderly people may be kept intact; the appendix with anatomical abnormalities (such as a long and curved appendix) has a higher risk of recurrent appendicitis, and appendectomy is recommended (6). In this article, because the child was young and at risk of recurrent appendicitis, appendectomy was performed. According to the literature, the prognosis for children with AH is generally favorable following appropriate surgical intervention. However, long-term follow-up is essential to monitor for potential complications such as recurrence or adhesions (15).

Laparoscopy, as a simple, accurate, rapid, and effective method for examining and evaluating the condition of the contralateral processus vaginalis, has been increasingly accepted by more and more surgeons in the treatment of inguinal indirect hernia in children. However, there are still few reports on its application in AH. For AH, through our preliminary attempt, laparoscopic appendectomy and high ligation of the hernial sac (or the unclosed processus vaginalis) are safe, effective, and feasible. With regard to the routine indication for contralateral inguinal exploration in cases of AH, current guidelines differ: some recommend routine exploration for unilateral inguinal hernia in children to avoid secondary surgery, while others caution against unnecessary exploration due to risks of spermatic cord injury or hydrocele. Individualized decisions should be based on intraoperative findings (16). However, this report only includes one case, and more research is needed to evaluate the safety of this approach.

## Data Availability

The raw data supporting the conclusions of this article will be made available by the authors, without undue reservation.
